# Epidemiology of Leprosy in Spain: The Role of the International Migration

**DOI:** 10.1371/journal.pntd.0004321

**Published:** 2016-03-03

**Authors:** José M. Ramos, David Romero, Isabel Belinchón

**Affiliations:** 1 Department of Internal Medicine, Hospital General Universitario de Alicante, Alicante, Spain; 2 Department of Medicine, Miguel Hernández University of Elche, Alicante, Spain; 3 Service of Dermatology, Hospital General Universitario de Alicante, Alicante, Spain; RTI International, UNITED STATES

## Abstract

**Background:**

Although incidence of leprosy in Spain has declined steadily over the years, the fivefold increase in immigration since the turn of the century—much of it from countries where leprosy is still prevalent—has been linked to an uptick in registered cases.

**Objective:**

To describe the epidemiologic trends of incident leprosy cases detected in Spain among Spanish- and foreign-born population groups.

**Methods:**

Observational, retrospective study of suspected leprosy cases in Spain, as reported through the System of Compulsory Notification of Diseases from 2003 to 2013, with results disaggregated by country of birth. We collected statistical data on leprosy burden for other countries from WHO to estimate the expected number of imported cases.

**Results:**

Of the 168 leprosy cases registered during the study period, 40 (24.6%) were in Spanish patients, while 128 (76.2%) were detected in legally resident immigrants. We identified a significantly higher number of imported leprosy cases during the 2008–2010 and 2011–2013 trienniums compared to the reference biennium 2003–2004 (OR 5.38, 95% CI 1.83–14.88 and OR 4.80, 95% CI 1.41–16.33, respectively). Most imported cases were diagnosed in Latin American immigrants (71.9%), especially Brazilians, but also Paraguayans, Bolivians and other nationalities from South and Central America. However, registered incidence was lower than expected for each year. For example, in 2003, the expected new cases in immigrants was 47.12, compared to only four cases that were actually detected (a 91% difference). Likewise, we expected to find 49.6 incident cases among immigrants in 2009, but only 15 new cases were reported (60% fewer than expected).

**Conclusion:**

Imported cases of leprosy are responsible for most leprosy incidence in Spain, and we cannot rule out some under-diagnosis. Clinicians should be made more aware of the potential for leprosy incidence among patients from countries where the disease is endemic.

## Author Summary

Most of cases of leprosy that are diagnosed in Europe come from other parts of the world. This study describes the epidemiologic trends of incident leprosy cases detected in Spain among Spanish- and foreign-born population groups from 2003 to 2013. We show that new cases of leprosy will continue to appear in the country regardless of the control measures taken at a domestic level, as people from leprosy-endemic areas will continue to migrate. That said, we also found that the actual number of incident cases registered through the national reporting system was far below the expected cases estimated from WHO statistical data on countries of origin. Nevertheless, leprosy should be considered among the differential diagnoses in patients presenting in Spanish health centres with suspected cutaneous and neurological signs and symptoms, especially if they are from Brazil, Paraguay, Bolivia or other areas where leprosy is prevalent. Given the high proportion of Latin American immigrants in Spain, our findings are very relevant, and it is advisable that the Spanish Medical University Programme reinstate the inclusion of leprosy in training for junior doctors.

## Introduction

Leprosy, also known as Hansen disease, is an infection caused by the bacterium *Mycobacterium leprae*. It affects the skin and peripheral nerves and may lead to pronounced functional limitations. The last few decades have seen a notable decline in leprosy incidence worldwide, due in part to general socioeconomic development as well as the advent of fixed-dose combination therapy. Globally, the number of cases of leprosy has decreased from 752,417 in 2000 to 180,618 in 2013 [1,2], with the overwhelming majority of cases in 2013 occurring in low- and middle-income countries: 71% in the WHO Region of South-East Asia, 15.5% in the Americas, 8.8% in Africa, 3.3% in the Western Pacific, and 1.2% in the Eastern Mediterranean [1].

In Spain, leprosy incidence has also abated, with an estimated incidence of 0.16 cases by 100,000 habitants in the second half of the twentieth century [3]. Economic growth and development (i.e., rising GDP) has had a large role to play in the declining incidence of leprosy [3]. However, this development—and the corollary reduction in leprosy cases—has not occurred at the same pace in every country, and differential levels of socioeconomic development between Spain and the developing world have also sparked a change in migratory flows. From 2000 to 2013, immigration to Spain quintupled, with many people coming from countries where leprosy is still prevalent [4,5]. In fact, most incident cases of leprosy in Spain, as reported in indexed medical journals, were diagnosed in foreign-born citizens from countries where leprosy is endemic, such as Brazil [6–8].

Although France does not have an official leprosy register, a recent epidemiologic study of leprosy in Metropolitan France in 2009 and 2010 showed 39 new cases [9]. On the other hand, in Italy there is an official leprosy register, the National Reference Centre for Hansen Disease. A recent report from that country showed a gradual reduction of leprosy cases in patients born in that country (with only 12 cases diagnosed between 1990 and 2009, the last of which was in 2003) but also a concurrent increase in imported incidence (n = 159) [10]. In Spain, the State Register of Leprosy began operating in 1992 under the direction of the National Epidemiology Centre. Based on a Case Report Sheet, the Register publishes a final report every year in the Weekly Epidemiological Bulletin [11,12].

For our study, we performed a retrospective analysis to characterise the trend of new leprosy cases registered in Spain, along with the prevalence and trends of cases occurring in patients born in Spain and elsewhere. We also compared the real incidence registered with the expected number of cases, as calculated based on the disease burden in origin countries. To our knowledge, this is the first such study of its kind in Spain, although we adopted a very similar methodology to the one used by Massone et al. in Italy [10], classifying the reported cases of leprosy by country of origin in citizens and legal residents of Spain from 2003 to 2013.

## Materials and Methods

Observational, retrospective study on incident leprosy cases reported through the National System of Compulsory Disease Notification, or SCDN (in Spanish, the Sistema de Enfermedades de Declaración Obligatoria) from 2003 to 2013.

In Spain, available data on leprosy were based on suspected new cases reported through the SCDN. The State Leprosy Register began operating in 1992 under the direction of the National Epidemiology Centre (part of the Carlos III Health Institute) as a result of collaboration between the Ministry of Social Affairs, the Ministry of Health, and the Autonomous Communities. Register data is collected using a Case Report Sheet and the Manual of Procedure, in accordance with WHO definitions, classifications and recommendations. Upon the creation of the National Epidemiologic Surveillance Network, leprosy was included among the diseases reported annually by special systems (i.e., by registration), with the relevant regulations stipulating that only active cases would be monitored at country level. We retrieved leprosy statistics for new cases in Spain between 2003 and 2013 from the State Leprosy Register.

We gathered official data on legally resident foreigners and their countries of origin from the Spanish National Statistics Institute (http://www.ine.es/). This data excludes undocumented immigrants, so the total number of foreign nationals residing in the country is unknown.

To estimate the expected number of leprosy cases, we collected statistics for the main countries of origin from the WHO Weekly Epidemiological Record (WER) for the period 2003–2013 [1, 13–23]. For the year 2002, we also have Spanish leprosy statistics, although these were unavailable from WHO.

We used the World Development Indicators and the online databases of the World Bank to collect population data from immigrants’ countries of origin [24], and we entered this data, together with Spanish leprosy statistics, world leprosy statistics, and data on the number of legal immigrants in Spain into a spreadsheet using Microsoft Excel 2011. We performed separate analyses for each year and country of origin to calculate expected cases and reported cases of leprosy in Spain.

We report the number of leprosy cases detected from 1 January to 31 December of a given year, as well as the patients’ countries or origin. Using 2003–2004 as the reference biennium, we calculated the trend of the number of reported cases in 2005–2007, 2008–2010 and 2011–2013. We calculated the odds ratio (OR) with 95% confidence interval (CI) and the case reported ratio by dividing the number of yearly cases reported in a given country by that country’s population, and multiplying that number by 100,000. For example, 33,955 cases were reported in Brazil in 2011, out of a population of 196,935,134, so the case reported ratio for that year was 17.24.

We then estimated the number of expected imported cases by multiplying the case reported ratio of each country by the number of legally resident immigrants in Spain from that country, and dividing the figure by 100,000. Thus, to calculate the expected number of cases imported from Brazil for 2011, we multiplied the case reported ratio by 107,596 (the number of Brazilians registered in Spain in 2011) and divided the result by 100,000, leaving us with an expected number of imported cases of 18.55.

Following the methodology of Massone et al. [10], we included in our analyses all of the countries of origin for immigrants diagnosed with leprosy in Spain between 2003 and 2013 and countries from which a certain number of cases could reasonably be expected, even if no immigrants from there had received a diagnosis during the study period. We defined this second group as countries that either reported more than 1000 leprosy cases per year or had at least 200 legally residents in Spain as well as one of the following characteristics in the study period: (a) >100 leprosy cases reported per year; or (b) new case detection rate > 1⁄100 000 population.

From there, we calculated a total case reported ratio from the total number of leprosy cases reported in the country of origin, divided by the total residents in Spain from that country, and multiplied by 100,000. Finally, we calculated the number of expected imported cases in Spain using available data for legal residents.

### Ethics Statement

The research protocol was approved by The Committee for Security of Information and Research at the Hospital General Universitario Alicante. This is an observational retrospective study from national NLDs and reported through SCDN. All analysed data were anonymised.

## Results

**Fig 1:**
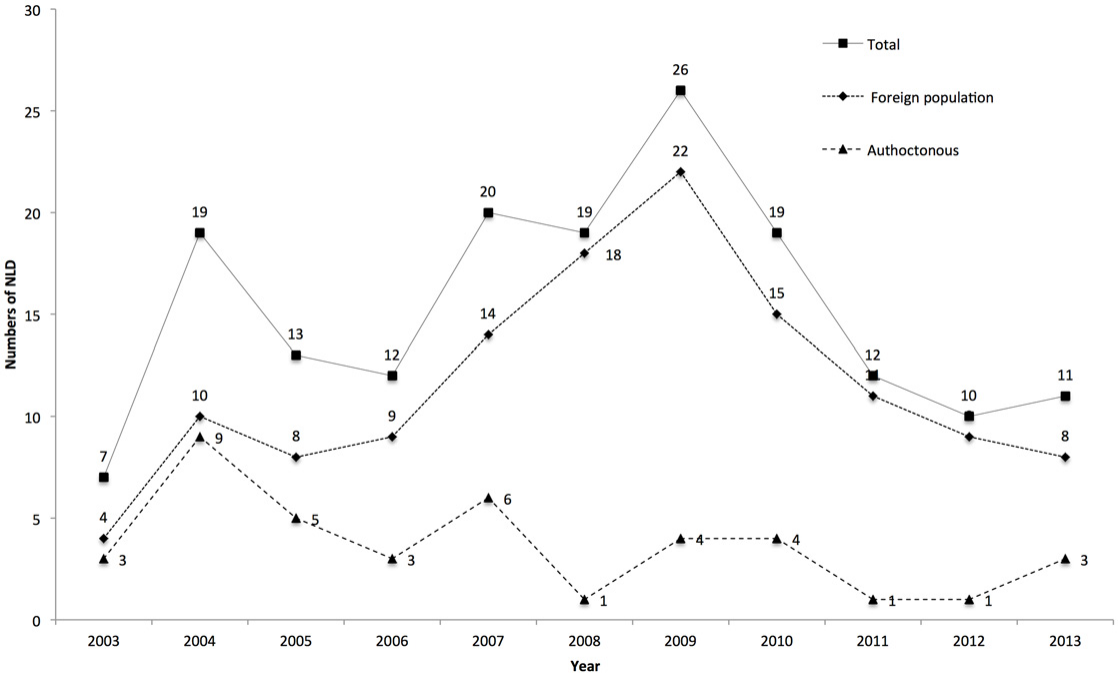
Trends of new leprosy cases detected during study period (2003–2013) in total, in foreign and autochthonous Spanish populations.

One hundred sixty-eight leprosy cases were reported in the study period: 40 (24.6%) in Spanish-born citizens and 128 (76.2%) in foreign nationals. Fig 1 shows the number of leprosy cases reported in years 2003–2013. Registered incidence in foreign nationals living in Spain was much higher in the 2008–2010 and 2011–2013 trienniums compared to the reference biennium 2003–2004 (OR 5.38, 95% CI 1.83–14.88 and OR 4.80, 95% CI 1.41–16.33) (Table 1). Of twenty different countries of origin analysed, the most cases of leprosy were reported in Latin Americans (71.9%), especially Brazilians, with a significant proportion also occurring among Paraguayans and Bolivians. The second most important region of origin was Africa (26.6%), particularly Morocco. Asian immigrants accounted for the fewest cases of leprosy, although among the cases registered, the greatest proportion was in citizens from the Phillipines and India. We could not identify the nationality of the immigrants in two cases (Table 2).

**Table 1:** Trends of new leprosy cases reported in foreign and Spanish-born citizens per two- or three-year period.

Periods	Foreign citizens		Spanish-born citizens		OR (95% CI)
	N	%	N	%	
2003–2004	14	53.8	12	46.2	1
2005–2007	31	68.9	14	31.1	1.90 (0.70–5.14)
2008–2010	55	85.9	9	14.1	5.38 (1.83–14.88)
2011–2013	28	84.8	5	15.2	4.80 (1.41–16.33)

OR = Odds ratio; 95% confidence intervals

**Table 2:** Regions and countries of the 128 leprosy cases in foreign citizen in the study period.

Country/Region	N	%
***Americas***	**92**	**71.9**
*Brazil*	41	320
*Paraguay*	18	14.1
*Bolivia*	13	10.2
*Ecuador*	8	6.3
*Colombia*	5	3.9
*Venezuela*	3	2.3
*Dominican Republic*	3	2.3
*Puerto Rico*	1	0.8
***Africa***	**34**	**26.6**
*Morocco*	7	5.5
*Senegal*	5	3.9
*Equatorial Guinea*	4	3.1
*Mali*	4	3.1
*Mauritania*	2	1.6
*Gambia*	1	0.8
*Nigeria*	1	0.8
*Burkina Faso*	1	0.8
*Congo*	1	0.8
*Somalia*	1	0.8
***Asia***	7	5.5
*Philippines*	4	3.1
*India*	3	2.3
***Not available***	2	1.6

We also included another 17 countries in our analyses even though no immigrants from there received a leprosy diagnosis between 2003 and 2013, since more than 1000 leprosy cases are reported in those countries every year, and a consistent number of cases could reasonably be expected (Table 3). The countries in this group were mainly in Africa and Asia, and together, they accounted for about 308,000 legal residents in Spain in 2013. Another 14 countries (Table 3) had a case reported ratio in the country of origin of ≥ 1 per 100,000 population between 2003 and 2013 or at least 100 leprosy cases reported each year between 2003 and 2013 and were considered eligible because of the high number of immigrants residing in Spain (always >200 per year). The population group in Spain originating in these countries was over 232,000 in 2013.

**Table 3:** Leprosy- endemic countries with more than 1000 new leprosy cases reported that had no immigrants diagnosed with leprosy in Spain, and countries with a ratio of case reported per 100,000 people in the country of origin ≥ 1 or with more than 100 case reported each year between 2003 and 2013 and living >200 per country.

Leprosy-endemic countries with more than 1000 case reported that had no foreign citizen diagnosed with leprosy in Spain	Countries with a ratio of case reported per 100,000 population in country of origin ≥ 1 or with more than 100 case reported each year
***Americas***	
Argentina	Cuba
	Mexico
	United Sates of America
***Africa***	
Angola	Cameroon
Cote d´Ivoire	Congo
Democratic Republic of the Congo	Ghana
Ethiopia	Guinea
Egypt	Kenya
Madagascar	Liberia
Mozambique	Sierra Leona
Tanzania	Togo
Sudan	
South Sudan	
***Asia***	
Bangladesh	Pakistan
China	Vietnam
Indonesia	Thailand
Myanmar	
Nepal	
Sri Lanka	

Table 4 shows that the imported cases reported were lower than expected for each year. In 2003, the expected number of leprosy cases in foreign nationals was 47.12, but there were only 4 cases reported (a difference of 91%). In 2010, there were 15 leprosy cases diagnosed in foreign-born residents, while the estimate for expected cases was 49.6 (a difference of 60%). Finally, in 2013, there were 8 leprosy cases in foreign residents, compared to 34 expected (a difference of 76%).

**Table 4:** New leprosy cases reported, estimated leprosy cases in foreign citizens, ratio of cases reported in Spain and in the countries of origin of foreign citizens between 2003 and 2013.

* *	2003	2004	2005	2006	2007	2008	2009	2010	2011	2012	2013
*Leprosy cases reported in Spain*	7	19	13	12	20	19	26	19	12	10	11
*Case reported ratio in Spain*	0.016	0.044	0.029	0.027	0.044	0.041	0.056	0.040	0.025	0.021	0.023
*Leprosy cases reported in Spain in foreign citizens*	4	10	8	9	14	18	22	15	11	9	8
*Expected leprosy cases in foreign citizens in Spain*	47.1	35.9	35.9	41.6	41.2	52.5	55.4	49.5	43.2	42.5	34.1
*% Less cases reported in foreign citizens than estimated cases*	91.5	72.1	77.7	78.4	66.0	65.7	60.3	69.7	74.5	78.8	76.5
*Case reported ratio in Spain in foreign citizens*	0.15	0.33	0.21	0.22	0.31	0.34	0.39	0.26	0.19	0.16	0.14
*Total-leprosy cases in the countries of origin*	13.35	14.77	6.68	5.29	5.51	5.17	4.16	4.42	4.28	4.14	3.80
*Difference total-leprosy cases in countries of origin and case reported ratio in Spain in foreign citizens*	13.2	14.44	6.47	5.07	5.2	4.83	3.77	4.16	4.09	3.98	3.66
*Total- leprosy cases in countries of origin contributing to case reported in Spain*	7.67	5.36	4.28	4.53	4.39	3.92	3.34	3.36	3.68	3.16	3.37

If we compare the annual number of leprosy cases in foreign residents in Spain and the total case reported ratio of the countries of origin, we find a difference of 13.2 more cases per 100,000 inhabitants diagnosed in the countries of origin in 2003 and 3.66 in 2013.

In Table 5 we detail the relevant characteristics of twenty immigrant populations in Spain, including the number of legal residents in Spain, the case reported ratio in their country of origin, the case reported ratio in Spain, and the expected leprosy cases in foreign citizens from 2003 to 2013. The highest number of cases of leprosy diagnosed in Spain was in Brazilians; in fact, between 2 and 11 cases have been diagnosed every year since 2004. Over the course of our study period, the number of legal residents from Brazil increased, and the case reported ratio in Brazil decreased (from 30.39 in 2003–2005, to 20.97 in 2006–2009, and 16.89 in 2010–2013). However, the difference between the case reported ratio in Brazil and the case reported ratio in Spain in Brazilian citizens (9.77 and 30.79, respectively, or an absolute difference of 21.02) decreased from 2003–2005 to 2010–2013 period (when these values were 15.34 and 16.84, respectively, an absolute difference of only 1.50). The difference between leprosy case reported in Brazilians in Spain (4 cases) and the expected leprosy cases in Brazilians (12 cases) in 2003–2005 also decreased (to 16 leprosy cases reported in Spain and 18 expected leprosy cases in Brazilians in 2010–2013); however, we did not find any other differences of note.

**Table 5:** New leprosy cases detected in Spain, Foreign population by country of origin, rate of case reported in Spain, and in their country of origin and estimated leprosy cases in foreign citizens by country in three periods.

*Country of origin*	2003–2005	2006–2009	2010–2013
***Brazil***			
*Cases reported in Spain*	4	21	16
*Population in Spain*	40944	101333	104277
*Ratio of cases reported in Spain*	9.77	20.72	15.34
*Ratio of cases in country*	30.39	20.97	16.84
*Expected cases in Spain*	12	21	18
***Paraguay***			
*Cases reported in Spain*	1	9	8
*Population in Spain*	8188	55944	86326
*Ratio of cases reported in Spain*	12.21	16.09	9.27
*Ratio of cases in country*	8.71	6.87	6.72
*Expected cases in Spain*	1	4	6
***Bolivia***			
*Cases reported in Spain*	1	8	4
*Population in Spain*	59575	203374	192992
*Ratio of cases reported in Spain*	1.68	3.93	2.07
*Ratio of cases in country*	1.34	1.11	n.a.
*Expected cases in Spain*	1	n.a.	n.a.
***Ecuador***			
*Cases reported in Spain*	2	4	2
*Population in Spain*	447265	434388	332992
*Ratio of cases reported in Spain*	0.45	0.92	0.60
*Ratio of cases in country*	0.64	0.64	n.a.
*Expected cases in Spain*	n.a.	2.78	n.a.
***Morocco***			
*Cases reported in Spain*	2	4	1
*Population in Spain*	436943	629171.25	777199
*Ratio of cases reported in Spain*	0.46	0.64	0.13
*Ratio of cases in country*	0.5	0.16	0.13
*Expected cases in Spain*	2	1	1
***Colombia***			
*Cases reported in Spain*	2	2	1
*Population in Spain*	266939	276984.	258676
*Ratio of cases reported in Spain*	0.75	0.72	0.39
*Ratio of cases in country*	2.24	1.02	0.82
*Expected cases in Spain*	6	3	2
***Senegal***			
*Cases reported in Spain*	1	3	1
*Population in Spain*	23253	43821	63424.75
*Ratio of cases reported in Spain*	4.30	6.85	1.58
*Ratio of cases in country*	3.73	2.41	n.a.
*Expected cases in Spain*	1	1	n.a.
***Philippines***			
*Cases reported in Spain*	0	1	3
*Population in Spain*	18454	22902	30095
*Ratio of cases reported in Spain*	0.00	4.37	9.97
*Ratio of cases in country*	3.45	2.57	2.02
*Expected cases in Spain*	1	1	1
***India***			
*Cases reported in Spain*	0	2	1
*Population in Spain*	14695	24193	34890
*Ratio of cases reported in Spain*	0.00	8.27	2.87
*Ratio of cases in country*	20.63	11.67	10.51
*Expected cases in Spain*	3	3	4
***Equatorial Guinea***			
*Cases reported in Spain*	3	0	1
*Population in Spain*	11936	14177	14611.75
*Ratio of cases reported in Spain*	25.13	0	6.84
*Ratio of cases in country*	n.a.	n.a.	n.a.
*Expected cases in Spain*	n.a.	n.a.	n.a.
***Mali***			
*Cases reported in Spain*	1	3	0
*Population in Spain*	8116	18706	24387
*Ratio of cases reported in Spain*	12.32	16.04	0
*Ratio of cases in country*	4.27	n.a.	1.7
*Expected cases in Spain*	0.3	n.a.	0.4
***Venezuela***			
*Cases reported in Spain*	0	0	3
*Population in Spain*	39213	55631	58968
*Ratio of cases reported in Spain*	0.00	0	5.09
*Ratio of cases in country*	3.79	2.33	n.a.
*Expected cases in Spain*	1	1	n.a.
***Dominican Republic***			
*Cases reported in Spain*	1	0	2
*Population in Spain*	49799	73028	92046
*Ratio of cases reported in Spain*	2.01	0	2.17
*Ratio of cases in country*	2.33	1.71	1.35
*Expected cases in Spain*	1	1	1
***Mauritania***			
*Cases reported in Spain*	1	1	0
*Population in Spain*	7945	10076.75	11423.5
*Ratio of cases reported in Spain*	12.59	9.92	0
*Ratio of cases in country*	2.01	n.a.	n.a.
*Expected cases in Spain*	n.a.	n.a.	n.a.
***Puerto Rico***			
*Cases reported in Spain*	0	1	0
*Population in Spain*	19508	20655	24414
*Ratio of cases reported in Spain*	0.00	4.84	0
*Ratio of cases in country*	n.a.	n.a.	n.a.
*Expected cases in Spain*	n.a.	n.a.	n.a.
***Gambia***			
*Cases reported in Spain*	0	1	0
*Population in Spain*	13954	18925	22029
*Ratio of cases reported in Spain*	0.00	5.28	0
*Ratio of cases in country*	n.a.	n.a.	1.97
*Expected cases in Spain*	n.a.	n.a.	0.43
***Nigeria***			
*Cases reported in Spain*	1	0	0
*Population in Spain*	21440	35876	45458
*Ratio of cases reported in Spain*	4.66	0	0
*Ratio of cases in country*	3.81	2.9	n.a.
*Expected cases in Spain*	0.82	1.04	n.a.
***Somalia***			
*Cases reported in Spain*	1	0	0
*Population in Spain*	n.a.	n.a.	n.a.
*Ratio of cases reported in Spain*	n.a.	n.a.	n.a.
*Ratio of cases in country*	2.59	2.78	n.a.
*Expected cases in Spain*	n.a.	n.a.	n.a.
***Burkina Faso***			
*Cases reported in Spain*	0	1	0
*Population in Spain*	401	797.5	1226
*Ratio of cases reported in Spain*	0.00	125.39	0
*Ratio of cases in country*	7.58	3.85	n.a.
*Expected cases in Spain*	0.03	0.03	n.a.

n.a.: not available

## Discussion

The prevalence of leprosy is declining, in part due to the advent of fixed-dose combination therapy [1]. Over 95% of the global leprosy burden is concentrated in nine countries, but most especially in India, Brazil, Madagascar, Mozambique and Nepal [2]. The increased migratory flow of people from areas where leprosy is still endemic into Europe, Northern America, Japan and Australia will undoubtedly have an impact on the incidence of leprosy in countries where leprosy has been considered eradicated or controlled for decades [10].

In Europe, most cases of leprosy are imported. For example, in Italy, Massone et al. observed a gradual reduction in leprosy cases among Italian-born citizens, where only 12 cases were registered between 1990 and 2009 (the last in 2003). However, they also observed a concurrent increase in cases imported from other countries (n = 159) [10]. Likewise, a recent epidemiologic study of leprosy in Metropolitan France, which took place in 2009 and 2010, found that out of 39 new cases, only 7 (18%) occurred in patients of French origin (2 from Metropolitan France and 5 from Overseas France) [9]. Another study also identified several cases of leprosy in France in foreign patients [25], and one described the case of a Portuguese woman living in France [26]. In 2000, Sequeira et al. reported that half of the incident cases of leprosy diagnosed in a dermatological clinic in Coimbra, Portugal were in foreigners, mainly from Brazil [27]. In our study, the number of cases in Spanish-born citizens is higher than in Italy or France. However, the proportion of new cases in Spanish-born citizens has decreased from 36.2% in 2003–2004 to 15.2% in 2011–2013. These cases occurred mostly in citizens residing in Spain (particularly in southern Spain) [7,28,29], although some lived in other countries [7]. However, it is also important to note that leprosy was endemic in both Spain [3] and Portugal [30] until relatively recently.

The long incubation period between the infection and clinical manifestation of leprosy may be a key factor explaining its importation, whereby asymptomatic people emigrate before developing any clinical signs of the disease [10]. Like Massone et al. [10], who conducted their study from 2003 to 2009, we found that the frequency of incident imported cases was lower than expected for each year. This discrepancy may be attributable to different factors, including the under-recognition and subsequent under-diagnosis of leprosy cases; the under-reporting of leprosy cases to the State Leprosy Register; or a lower level of exposure to the bacterium among the immigrant population than in the local populations in the country of origin. However, in some countries with available data, such as Paraguay, there were fewer reported cases of leprosy than the expected leprosy cases in Paraguayans in Spain, suggesting that under-reporting and under-diagnosing occurs in the country of origin as well as in the destination.

Our data are an incomplete measure of the leprosy burden in Spain, as we only recorded the nationally registered cases, which would not take into account any cases that were not reported or diagnosed. Moreover, the Spanish case reported ratio only corresponds to legal foreign residents from leprosy-endemic areas registered in the Spanish National Statistics Institute, not to undocumented immigrants. It was therefore impossible to calculate the total case reported ratio among all immigrants from leprosy- endemic areas.

Although there is a risk of secondary transmission of leprosy from foreign- to Spanish-born citizens, the State Register of Leprosy did not provide enough information to determine whether this was true for the Spanish-born cases in our study. However, in a series of cases of imported leprosy reported between 1978 and 1988 in the United States, investigators found no cases of transmission [31], so the impact of secondary transmission is likely to be limited.

It should also be noted that leprosy might mimic other diseases. Nery et al [32] studied uncommon presentations and delay in the diagnosis of leprosy in Rio de Janeiro (a leprosy-endemic area), recommending that clinicians working where leprosy is prevalent consider that disease in their differential diagnosis, especially if the clinical presentation is unusual, as this could facilitate the diagnosis of more cases. However, clinicians must also be capacitated to recognise leprosy in the non-endemic industrialised world [33]. General practitioners and dermatologists should be aware of leprosy’s symptoms and clinical manifestation, considering the diagnosis of the disease a real possibility, especially in patients coming from leprosy-endemic areas [10,32,33]. The leprosy patients in our study were diagnosed late in the course of the disease, when it is possible to present with leprosy reaction [8,34,35], all the more reason for clinicians to be familiar with an atypical presentation. In Spain, clinicians should include leprosy among the potential causes of cutaneous and neurological signs and symptoms, especially if patients are from Brazil, Paraguay, Bolivia or other areas where leprosy is prevalent [6,8,34,35]. This is particularly relevant because most of the immigrant population living in Spain is from Latin America. In other parts of Europe, where there are more immigrants from Sub-Saharan Africa and Asia, leprosy should be also be considered. Given our findings, it would be advisable for the Spanish Medical University Programme to reinstate teaching junior doctors about leprosy.

This study is one approach to evaluating how the problem of leprosy is being addressed in Spain. Our data are not comprehensive, but they do provide a preliminary indication of the importance of the leprosy burden in Spain. In a globalised world, even the tight control of leprosy cases originating in Spain will not prevent its future incidence in the country, as people will continue to enter Spain from countries where the disease is more common. Researchers from other European countries are encouraged to carry out studies similar to the present one and the one conducted by Massone et al. [10] in Italy, in order to better understand the leprosy situation in other parts of the continent.

## Supporting Information

S1 Checklist STROBE checklist. (DOCX)

## References

[1] Global leprosy update, 2013. WHO- Wkly Epidemiol Rec. 2014; 89: 389–400. 25202781

[2] Leprosy-global situation. Wkly Epidemiol Rec. 2000;75: 226–31. 10920713

[3] Alfonso JL, Vich FA, Vilata JJ, de las Aguas JT. Factors contributing to the decline of leprosy in Spain in the second half of the twentieth century. Int J Lepr Other Mycobact Dis. 2005; 73: 258–68. 16830635

[4] Instituto Nacional de Estadística: Cifras de Población a 1 de enero de 2013 –Estadística de Migraciones 2012. Available at: http://www.ine.es/prensa/np788.pdf (accessed 2 November 2014).

[5] Instituto Nacional de Estadística. Cifras de Población a 1 de julio de 2013 Estadística de Migraciones. Primer semestre de 2013 Available at: http://www.ine.es/prensa/np822.pdf (last accessed 2 November 2014).

[6] Biosca G, Casallo S, López-Vélez R. Methotrexate treatment for type 1 (reversal) leprosy reactions. Clin Infect Dis. 2007; 45: e7–9. 17554691 10.1086/518699

[7] Contreras-Steyls M, López-Navarro N, Herrera-Acosta E, Castillo R, Ruiz del Portal G, Bosch RJ, Herrera E. The current challenge of imported leprosy in Spain: a study of 7 cases. Actas Dermosifiliogr. 2011;102:106–13. 10.1016/j.ad.2010.10.008 21334586

[8] de Guzmán MT, Cortés I, Pedro Zabaleta J, Antonio Aramburu J. A male from Brazil presenting skin lesions and fever. Enferm Infecc Microbiol Clin. 2009; 27: 422–4. 10.1016/j.eimc.2009.01.012 19419800

[9] Bret S, Flageul B, Girault PY, Lightburne E, Morand JJ. Epidemiological survey of leprosy conducted in metropolitan France between 2009 and 2010. Ann Dermatol Venereol. 2013; 140: 347–52. 10.1016/j.annder.2013.02.019 23663706

[10] Massone C, Brunasso AM, Noto S, Campbell TM, Clapasson A, Nunzi E. Imported leprosy in Italy. J Eur Acad Dermatol Venereol. 2012; 26:999–1006. 10.1111/j.1468-3083.2011.04201.x 21831112

[11] Rodríguez E, Díaz O. Vigilancia de la lepra en España en 2013 y situación mundial. Bol Epidemiol Semanal. 2014; 22: 34–42.

[12] Rodríguez E, Díaz O, Hernández G. Vigilancia de la lepra. Situación en el mundo y España 2011. Bol Epidemiol Semanal 2012; 20:17–25.

[13] Global leprosy situation, 2004. Wkly Epidemiol Rec. 2005; 80: 118–24. 15875655

[14] Global leprosy situation, 2005. Wkly Epidemiol Rec 2005; 80: 289–96. 16149384

[15] Global leprosy situation, 2006. Wkly Epidemiol Rec 2006; 81: 309–16. 16903018

[16] Global leprosy situation, 2007. Wkly Epidemiol Rec 2007, 82: 225–32. 17585406

[17] Global leprosy situation, beginning of 2008. Wkly Epidemiol Rec. 2008; 83: 293–300. 18705152

[18] Global leprosy situation, 2009. WHO–Wkly Epidemiol Rec 2009; 84: 333–40. 19685606

[19] Global leprosy situation, 2010. WHO–Wkly Epidemiol Rec 2010; 85: 337–48. 20830851

[20] Leprosy update, 2011. WHO- Wkly Epidemiol Rec 2011; 86: 389–400. 21887885

[21] Global leprosy situation, 2012. WHO- Wkly Epidemiol Rec 2012; 87: 317–28. 22919737

[22] Global leprosy: update on the 2012 situation. WHO- Wkly Epidemiol Rec 2013; 88: 365–80. 24040691

[23] Global leprosy update, 2013.Wkly Epidemiol Rec 2014; 89: 389–400. 25202781

[24] World Development Indicators. Available at: http://data.worldbank.org/data-catalog/world-development-indicators (last accessed 7 November 2014).

[25] Gain M, Ghnaya H, Lepeytre F, Toledano C, Cabane J, Phong Tiev K. Leprosy: a rare imported disease. Rev Med Interne. 2009; 30: 1064–6. 10.1016/j.revmed.2009.03.010 19836114

[26] Ezzedine K, Malvy D, Beylot C, Longy-Boursier M. Autochthonous leprosy in metropolitan France presenting with a diffuse infiltration of the face and febrile illness. Int J Dermatol. 2009; 48: 69–72. 10.1111/j.1365-4632.2009.03831.x 19126055

[27] Sequeira J, Martins C, Marques C, Machado A, Baptista AP. Leprosy. Comparative study of old and new patients. Acta Med Port. 2000; 13: 13–7 11059050

[28] Serrano-Pozo A, Gómez-Aranda F, Giles M, Chinchón D, Chinchón I, Bautista-Lorite J. Sensory polyneuropathy as initial manifestation of endemic leprosy in Spain. Eur Neurol. 2004;52:256–8. 15583462 10.1159/000082374

[29] Pardal-Fernández JM1, Rodríguez-Vázquez M, Fernández-Aragón G, Iñíguez-De Onzoño L, García-Muñozguren S. Leprosy and severe neuropathy in two native Spaniards. Rev Neurol. 2007;45:734–8. 18075988

[30] Medeiros S, Catorze MG, Vieira MR. Hansen's disease in Portugal: multibacillary patients treated between 1988 and 2003. J Eur Acad Dermatol Venereol. 2009; 23: 29–35. 10.1111/j.1468-3083.2008.02941.x 18713227

[31] Mastro TD, Redd SC, Breiman RF. Imported leprosy in the United States, 1978 through 1988: an epidemic without secondary transmission. Am J Public Health. 1992; 82: 1127–30 1636833 10.2105/ajph.82.8.1127 PMC1695739

[32] Nery JA, Schreuder PA, de Mattos PC de Mendonça LV, Tardi RT, de Mello S et al Hansen’s disease in a general hospital: uncommon presentations and delay in diagnosis. J Eur Acad Dermatol Venereol. 2009; 23: 150–6. 10.1111/j.1468-3083.2008.03006.x 18785893

[33] Forno C, Häusermann P, Hatz C, Itin P, Blum J. The difficulty in diagnosis and treatment of leprosy. J Travel Med. 2010; 17: 281–3. 10.1111/j.1708-8305.2010.00419.x 20636605

[34] Aranegui B1, Abalde T, Peón G, Alvarez-Álvarez C, de la Torre C. Lucio's phenomenon after childbirth. Int J Dermatol. 2014;53:e127–9. 10.1111/j.1365-4632.2012.05634.x 23557094

[35] Camps-García T, Pedro IP, Gómez IM, Narankiewicz D, Ayala-Gutierrez M, Sanz-Trelles A. Clinical images: cutaneous necrotizing vasculitis in a patient with lepromatous leprosy. Arthritis Rheum. 2011;63:3639 10.1002/art.30602 22038407

